# Pulmonary Hypertension and Hypocholesterolemia Secondary to Thyrotoxicosis

**DOI:** 10.1155/2020/8884061

**Published:** 2020-11-12

**Authors:** Narangoda Liyanage Ajantha Shyamali, Chandrike Ponnamperuma

**Affiliations:** ^1^University of Sri Jayewardenepura, Gangodawila, Nugegoda, Sri Lanka; ^2^National Hospital of Sri Lanka, Colombo 10, Sri Lanka

## Abstract

**Background:**

Thyroid disorders commonly affect the cardiovascular system. Thyrotoxicosis leading to pulmonary hypertension has been increasingly reported during recent years. Thyroid dysfunction affects the lipid metabolism, and thyrotoxicosis can be associated with low lipid levels. Thyrotoxicosis presenting with right ventricular dysfunction is rare, and only few cases had been reported. *Case Presentation*. A 53-year-old woman presented with progressive shortness of breath and swelling of body for four months. Examination showed generalized oedema and a systolic murmur over the left sternal border. Transthoracic echocardiography confirmed pulmonary hypertension with tricuspid regurgitation. Investigations revealed thyrotoxicosis and very low cholesterol levels. Diagnosis of Graves' disease was confirmed with detection of thyrotropin receptor antibodies. Pulmonary pressure was normalized six months after antithyroid therapy.

**Conclusion:**

Thyrotoxicosis is a recognized cause of reversible pulmonary hypertension and acquired hypocholesterolemia. However, most clinicians are not aware of these associations. This case illustrates the importance of assessing thyroid function in patients presenting with pulmonary hypertension.

## 1. Introduction

Thyrotoxicosis is well known to associate with left ventricular dysfunction due to long-standing effects of thyroid hormones. However, the association of hyperthyroidism with right ventricular dysfunction secondary to pulmonary hypertension has been reported in the literature since 1980 [1]. The exact mechanism of pulmonary hypertension in thyrotoxicosis is ill-defined [2, 3]. Several studies have reported the resolution of pulmonary hypertension secondary to thyrotoxicosis with antithyroid therapy [4]. The association of hypothyroidism with hyperlipidemia has been well known for a long period. However, low cholesterol level secondary to thyrotoxicosis is not widely appreciated. Only few cases of Graves' thyrotoxicosis with pulmonary hypertension and hyperlipidemia have been reported in the literature.

## 2. Case Presentation

A 53-year-old previously healthy woman presented with fever for 3 days associated with a four-month history of progressive shortness of breath and generalized body swelling. She has also noted increased sweating which she considered as a perimenopausal symptom. On examination, she was febrile with a heart rate of 101/min and oxygen saturation of 98% on room air. She had bilateral ankle oedema and extensive abdominal wall oedema. Jugular venous pressure was elevated, and there was a pansystolic murmur over the left sternal border. Respiratory system examination was clinically normal.

**Figure 1:**
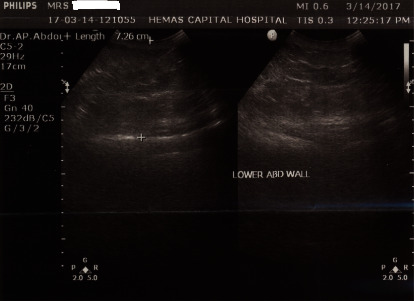
Ultrasound scan of the abdomen showing extensive abdominal wall oedema.

**Figure 2:**
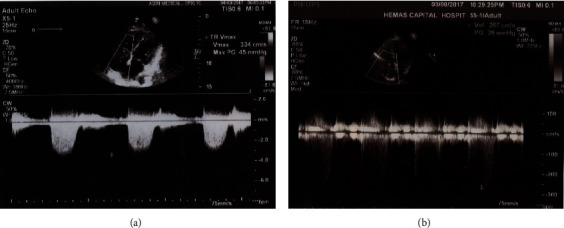
Transthoracic echocardiography demonstrating pulmonary artery pressures before (a) and after (b) the treatment of thyrotoxicosis.

Her basic blood investigations of full blood count, erythrocyte sedimentation rate, C-reactive protein levels, renal function tests, and liver function tests were normal. She was found to have low cholesterol levels with total cholesterol of 61 mg/dl (normal range: 140–239), low-density lipoprotein cholesterol (LDLC) of 26 mg/dl (normal range: 75–159), and high-density lipoprotein cholesterol (HDLC) of 20 mg/dl (normal range: 35–85). Thyroid-stimulating hormone (TSH) level was 0.004 *µ*IU/ml (normal range: 0.4–4.0) with free thyroxin (FT4) 5.48 ng/dl (normal range: 0.89–1.76) and free triiodothyronine (FT3) 11.48 pg/dl (normal range: 1.5–4.1). TSH receptor antibodies were positive with 4.39 IU/L (normal < 2 IU/L). Ultrasound of the thyroid showed diffusely enlarged glands with increased vascularity, and ultrasound scan of the abdomen revealed marked lower abdominal wall oedema without organomegaly (Figure 1). Transthoracic echocardiography showed right ventricular enlargement with tricuspid regurgitation. The systolic pulmonary artery pressure gradient was 45 mmHg (normal < 25 mmHg) (Figure 2(a)).

Graves' thyrotoxicosis was diagnosed, and she was started on antithyroid medication. She underwent computed tomographic pulmonary angiography (CT-PA) ten days after starting thyrostatic drugs which did not show evidence of chronic thromboembolic pulmonary hypertension or abnormalities of lung parenchyma. After six months of treatment, her symptoms improved. The systolic pulmonary artery pressure gradient was reduced to 29 mmHg in repeated transthoracic echocardiography (Figure 2(b)). TSH level increased to 0.48 µIU/ml with normalization of thyroid hormone levels. Her serum lipid levels returned to normal with total cholesterol of 185 mg/dl, LDLC of 109 mg/dl, and HDLC of 61 mg/dl.

## 3. Discussion

The association of thyrotoxicosis with right ventricular dysfunction was first reported in the literature in 1973 [5]. Thyrotoxicosis as a cause for pulmonary hypertension and reduction of pulmonary artery pressure with treatment of antithyroid medication have been clearly established recently [6, 7]. Therefore, most clinicians are not aware of this important association.

In a study of 23 patients with Graves' thyrotoxicosis, 65% of patients had pulmonary hypertension which normalized with definitive treatment, and a recent single-center cross-sectional study carried out in Ethiopia showed that 30% of patients with hyperthyroidism have echocardiographic evidence of pulmonary hypertension [4, 8]. Although the underlying pathophysiology for this complication is unclear, several mechanisms have been hypothesized: endothelial injury secondary to high cardiac output, immune-mediated endothelial damage, and enhanced metabolism of intrinsic pulmonary vasodilators [3, 9].

Increased expression of low-density lipoprotein (LDL) receptors and activity of lipoprotein lipase by thyroid hormones decrease the circulating levels of lipoproteins. Consequently, decreased levels of HDLC and LDLC are seen in patients with hyperthyroidism [10, 11]. The action of thyroid hormones in the liver is responsible for the reduction of LDLC levels, which is caused by enhanced transcription of LDL receptors in the liver. Therefore, the extent of LDLC level reduction is proportional to free thyroid hormone levels [11]. Hyperthyroidism is recognized as a significant cause of acquired hypocholesterolemia and unanticipated improvement of lipid levels in hyperlipidemic patients [12].

Thyrotoxicosis is an overlooked, reversible cause of pulmonary hypertension, and there is a high prevalence of pulmonary hypertension among hyperthyroid patients [13]. Thus, patients presenting with thyrotoxicosis and dyspnea should be screened for pulmonary hypertension [14]. All clinicians should be aware of this association, and this case highlights the importance of assessing thyroid function tests in patients with pulmonary hypertension.

## Data Availability

The clinical details and results of investigations are documented in bed head tickets. Bed head tickets are available in the record room of Asiri Medical Private Hospital of Sri Lanka. All the original reports are with the patient.

## Abbreviations

LDLC: Low-density lipoprotein cholesterol
HDLC: High-density lipoprotein cholesterol
TSH: Thyroid-stimulating hormone
FT4: Free thyroxin
FT3: Free triiodothyronine.
